# Inhibition of GPR68 induces ferroptosis in diffuse intrinsic pontine gliomas

**DOI:** 10.3389/fonc.2026.1808752

**Published:** 2026-05-13

**Authors:** Leif R. Neitzel, Aaron H. Wasserman, Rintaro Hashizume, Charles H. Williams, Charles C. Hong

**Affiliations:** 1 Department of Medicine, Michigan State University College of Human Medicine, East Lansing, MI, United States; 2 Henry Ford Health + Michigan State Health Sciences, Detroit, MI, United States; 3 Department of Pediatrics, Division of Pediatric Hematology and Oncology, University of Alabama at Birmingham, Children’s of Alabama, Birmingham, AL, United States

**Keywords:** diffuse intrinsic pontine glioma, dipg, GPR68, OGM, ogremorphin

## Abstract

**Background:**

Diffuse Intrinsic Pontine Glioma (DIPG) is a highly aggressive pediatric brainstem tumor with limited treatment options and dismal prognosis. Previously, we showed that Ogremorphin (OGM), a specific GPR68 inhibitor, induced ferroptosis in glioblastoma multiforme (GBM) cells. Given their shared originating cell type, we investigated OGM’s efficacy in inducing ferroptosis in DIPG cells, using normal human astrocytes (NHAs) for comparison.

**Methods:**

DIPG cell lines (SF7761, DIPG36, DIPG007) and NHAs were evaluated. OGM sensitivity was assessed via CellTiter-Glo viability assays. Ferroptosis markers including glutathione (GSH) levels, lipid peroxidation (BODIPY C11), and qRT-PCR of ATF4 and SLC7A11 were measured. Rescue experiments used liproxstatin-1 for ferroptosis and Z-VAD-FMK for apoptosis. Genetic validation used shRNA knockdown and overexpression of GPR68/ATF4, plus an ATF4 luciferase reporter assay.

**Results:**

OGM treatment in N5 media revealed that NHAs were more resistant (LD_50_ = 3.84 µM) than DIPG cells (LD_50_ ≤ 0.79 µM). DIPG sensitivity to OGM was comparable to GBM. Knockdown of GPR68 via shRNA confirmed that GPR68 inhibition induces cell death in DIPG cells. OGM robustly triggered ferroptosis marked by elevated lipid radicals, reduced GSH, and increased *ATF4* and *SLC7A11* expression in DIPG and NHA cells, except SF7761, which lacked *SLC7A11* induction. Furthermore, liproxstatin-1 rescued the loss of cell viability, whereas Z-VAD-FMK had no effect. ATF4 overexpression caused cell death, while its knockdown rescued OGM-mediated effects.

**Conclusions:**

These findings suggest that GPR68 inhibition is an attractive therapeutic target for DIPG. Notably, GPR68 inhibition mirrors the effects observed in GBM, inducing ATF4-dependent ferroptotic cell death.

## Introduction

Diffuse intrinsic pontine glioma (DIPG) and Glioblastoma multiforme (GBM) are among the most lethal brain tumors (1, 2). DIPG has a median survival of 10–11 months, while GBM median survival is 14 months despite aggressive standard management strategies (1, 3). DIPGs account for ~10-20% of pediatric brain tumors with a peak age of incidence of 6–9 years and rank as the most aggressive primary brain tumor in this population (4–8). GBMs are the most prevalent and lethal primary brain tumors among adults (1, 3). GBM typically arises from glial cells within the cerebral hemispheres, whereas DIPG originates in the developing pons of children, reflecting distinct anatomical derivations. Current standard of care for DIPGs consists of palliative radiation, whereas GBM treatment involves maximal surgical resection followed by radiation and chemotherapy (3). Clinical trials for specific molecular targets in DIPG remain scarce. Similarly, the high molecular heterogeneity complicates the identification of therapeutic targets in GBMs (9, 10). However, recent work suggests the small molecule Ogremorphin (OGM) as a promising therapeutic agent *in vitro* and *in vivo*, demonstrating efficacy against lung and pancreatic cancer cell lines as well as 13 GBM lines (11–13).

OGM specifically inhibits GPR68, also known as ovarian cancer G protein-coupled receptor 1 (OGR-1) (12–14). GPR68 is a proton sensing GPCR activated in response to extracellular acidification between pH 7.4 (inactive) and 6.4 (active) (15). A distinctive feature of GBMs is the acidic extracellular tumor microenvironment (TME), driven by the Warburg effect - a hallmark of cancer that promotes cancer progression by fostering malignant clonal selection, metastasis, pro-oncogenic transcriptional responses, and immune escape (16–25). Emerging data implicates the Warburg effect in fueling DIPG pathogenesis, though supporting evidence remains sparse (26, 27). In GBMs, GPR68 suppresses ATF4 expression in a pro-survival pathway to protects against ferroptosis - iron-mediated programmed cell death (13). Moreover, GPR68 is upregulated in radioresistant cell lines and the acidic TME confers radio-resistance (28, 29). Inhibition of GPR68 in GBMs, lung and pancreatic cancer cell lines induces ferroptosis and augments radiosensitivity (11–13). To our knowledge, no published studies have explored GPR68-mediated ferroptosis in DIPGs. The potential for GPR68 inhibition in DIPGs to induce ferroptosis and/or sensitize tumors to radiotherapy makes OGM an attractive therapeutic agent. Herein we investigated how disruption of GPR68 in DIPGs induced ferroptotic cell death in an ATF4-dependent manner, echoing its role in GBMs (13). Taken together, these findings suggest an exciting new direction for the treatment of DIPGs.

## Results

### DIPG cells are sensitive to OGM

**Figure 1:**
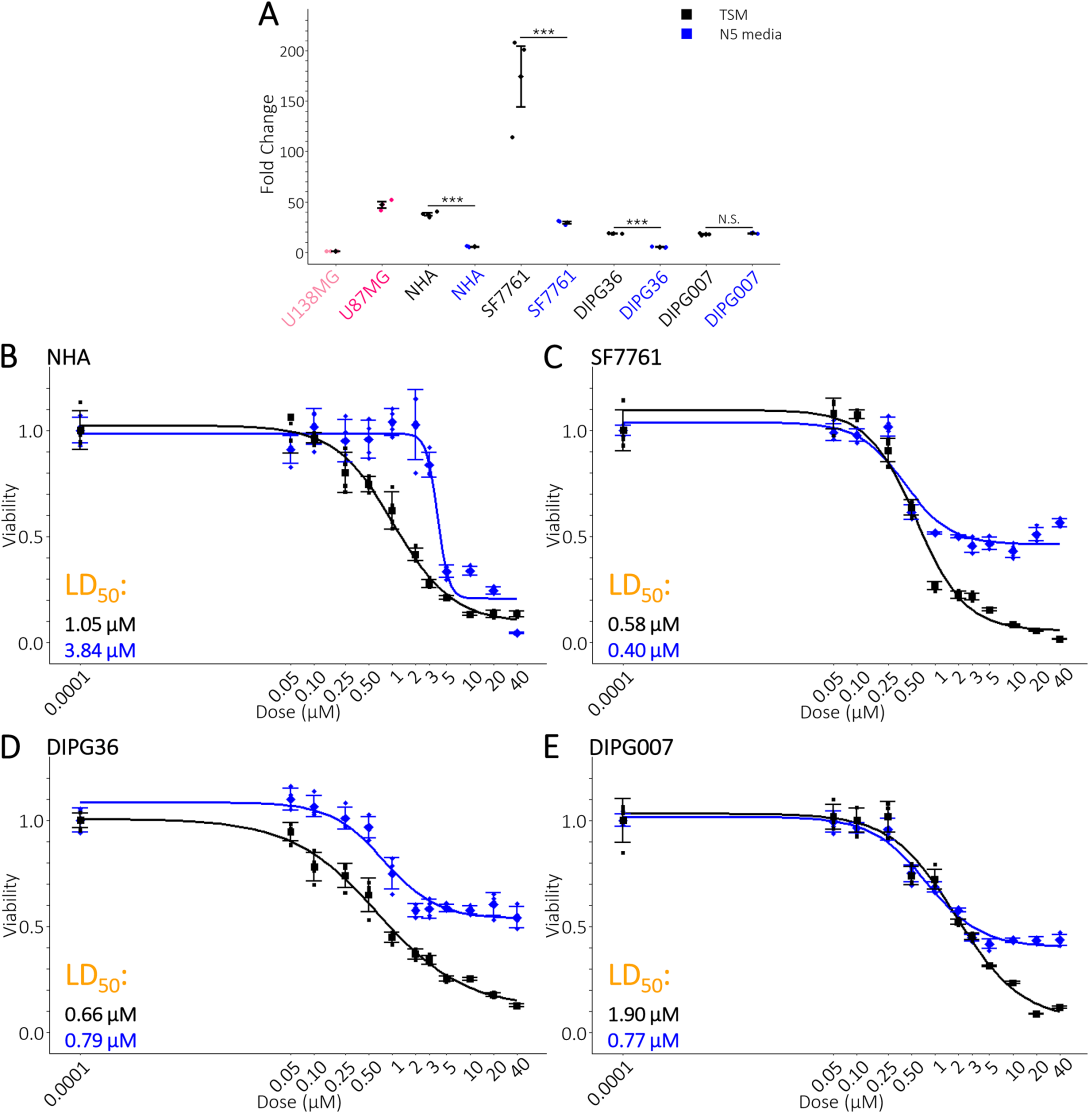
DIPG cells are sensitive to ogremorphin. **(A)** Expression of GPR68 in DIPG cells in mixed culture (TSM) or adherent culture (N5 media). Data are normalized to the U138MG glioblastoma cell line. **(B)** Four parameter logistic (4PL) regression of NHA cells in TSM (Black) and N5 media (Blue). The LD_50_ of NHA cells were lower in TSM than N5 media. **(C-E)** 4PL regression of three DIPG cell lines in TSM (Black) and N5 media (Blue). The LD_50_ of SF7761 and DIPG36 cells were relatively unchanged between mixed and adherent cultures. Adherent DIPG007 cells had a lower LD_50_ than mixed cell cultures. However, SF7761 and DIPG36 had lower viability at higher dosages. **(A)** *n* = 3 biological repeats with *n* = 3 technical repeats. **(B, E)** *n* = 3 biological repeats with *n* = 4 technical repeats. **(A)** mean +/- SD with significance determined by two-tailed, equal variance, t-tests. **(B-E)** mean +/- SD. **(A)** Comparison between TSM and N5 media. ***ρ<0.001.

We have shown OGM induces ferroptosis in GBM, lung carcinoma and pancreatic ductal adenocarcinoma cell lines (12, 13). Furthermore, zebrafish xenograft studies demonstrate that two GBM lines, U87MG and U138MG, are sensitive to OGM *in vivo (*11). Twelve of the thirteen tested GBM lines had an LD_50_ <2 µM (13). We therefore sought to test whether DIPGs are sensitive to OGM like GBMs. OGM is a specific inhibitor of GPR68, a receptor with neuroprotective properties; therefore, we compared DIPGs to Normal Human Astrocytes (NHA) (30). The DIPG cell lines SF7761, DIPG36, and DIPG007 can be grown as a mixed culture (adherent and suspension cells) in Tumor Stem Medium (TSM) or induced to become an adherent culture in N5 media (TSM + 5% FBS). NHA cells are adherent in both media types. We first sought to determine whether the expression of GPR68 depended on culture conditions (**Figure 1A**). GPR68 expression in NHA, SF7761, and DIPG36 decreased in N5 media. Conversely, expression in DIPG007 was unchanged. This media-dependent expression of GPR68 suggests that media may influence the response to OGM.

Therefore, we treated both mixed and adherent cultures with OGM to determine their LD_50_ (**Figures 1B-E**). NHAs in N5 media were more resistant to OGM, while DIPG007 cells were more sensitive (**Figures 1B,E**). The sensitivity of SF7761 and DIPG36 was comparable in both cultures (**Figures 1C,D**). SF7761, DIPG36, and DIPG007 exhibited higher survival in N5 media than in TSM at higher OGM concentrations (**Figures 1C-E**). This suggests that GPR68 expression does not correlate with sensitivity in DIPG cells. OGM may be promoting the conversion of adherent cells into suspension cells. To test this, we measured the number of live cells in suspension in N5 media and observed no change or a decrease in response to OGM (**Supplementary Figure 1A**). This demonstrates that OGM is killing the cells rather than inhibiting attachment. Furthermore, adherent DIPGs were sensitive to OGM at levels comparable to GBMs (LD_50_ ≤0.79 µM) while NHAs had a significantly higher LD_50_ (LD_50_ = 3.84 µM). Taken together, our data suggest DIPGs are as sensitive to OGM as GBMs.

### GPR68 knockdown kills DIPG cells

**Figure 2:**
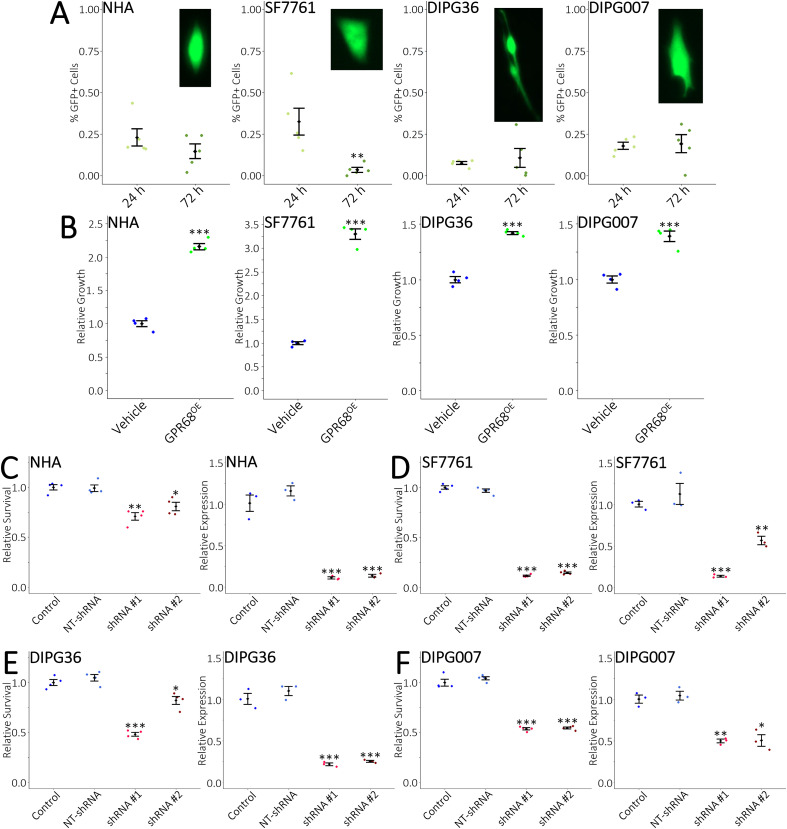
GPR68 expression is essential for DIPG cells. **(A)** Expression of EGFP in adherent NHA and DIPG cells demonstrates transfectability. Cells were evaluated at 24- and 72-hours post-transfection. **(B)** Overexpression of hGPR68 promotes the growth of adherent NHA and DIPG cells. **(C)** shRNA-mediated GPR68 knockdown in NHAs decreased survival (left). Decreased GPR68 expression was verified by qRT-PCR (right). **(D-F)** shRNA-mediated GPR68 knockdown in DIPGs decreased survival (left). Decreased GPR68 expression was verified by qRT-PCR (right). **(A)** *n* = 5 biological repeats with *n*≥1 technical repeats. **(B)** *n*≥3 biological repeats with *n* = 4 technical repeats. **(C-F)** *n* = 4 biological repeats with *n* = 4 technical repeats for survival, and *n* = 3 biological repeats with *n* = 3 technical repeats for qRT-PCR. **(A)** images not to scale. **(A, B)** mean +/- SD with significance determined by two-tailed, equal variance, t-tests with **ρ<0.01, ***ρ<0.001. **(C-F)** mean +/- SD with significance determined by multiple two-tailed, equal variance, t-tests with Bonferroni correction. Comparisons are between control and NT-shRNA, shRNA #1, or shRNA #2, with *ρ<0.01250, **ρ<0.00333, ***ρ<0.000333.

To verify if OGM was killing DIPG cells in a GPR68-specific manner we tested GPR68 using genetic methods. Knockdown of GPR68 with siRNA, shRNA, and CRISPRi has been shown to kill U87MG, U138MG, A549, and PANC02 cell lines (11–13). We first tested the transfectability of DIPGs with an EGFP-expressing plasmid (**Figure 2A**). A decrease in EGFP+ cells between 1- and 3- days post-transfection was observed in SF7761 cells (**Figure 2A**). This suggests they may not retain plasmids for long. Overexpression of human GPR68 in NHAs and DIPGs resulted in increased growth as assessed by CellTiter-Glo (**Figure 2B**). GPR68 overexpression did not promote the conversion of adherent cells into suspension cells (**Supplementary Figure 1B**). This is consistent with previous observations in A549 and PANC02 cell lines (12). Conversely, GPR68 knockdown with shRNA resulted in cell death (**Figures 2C-F**). As with OGM, the number of live cells in suspension in the N5 media was unchanged or decreased (**Supplementary Figure 2A**). These data suggest GPR68 inhibition is lethal to NHA and DIPG cells.

### OGM induces ferroptosis in DIPGs

**Figure 3:**
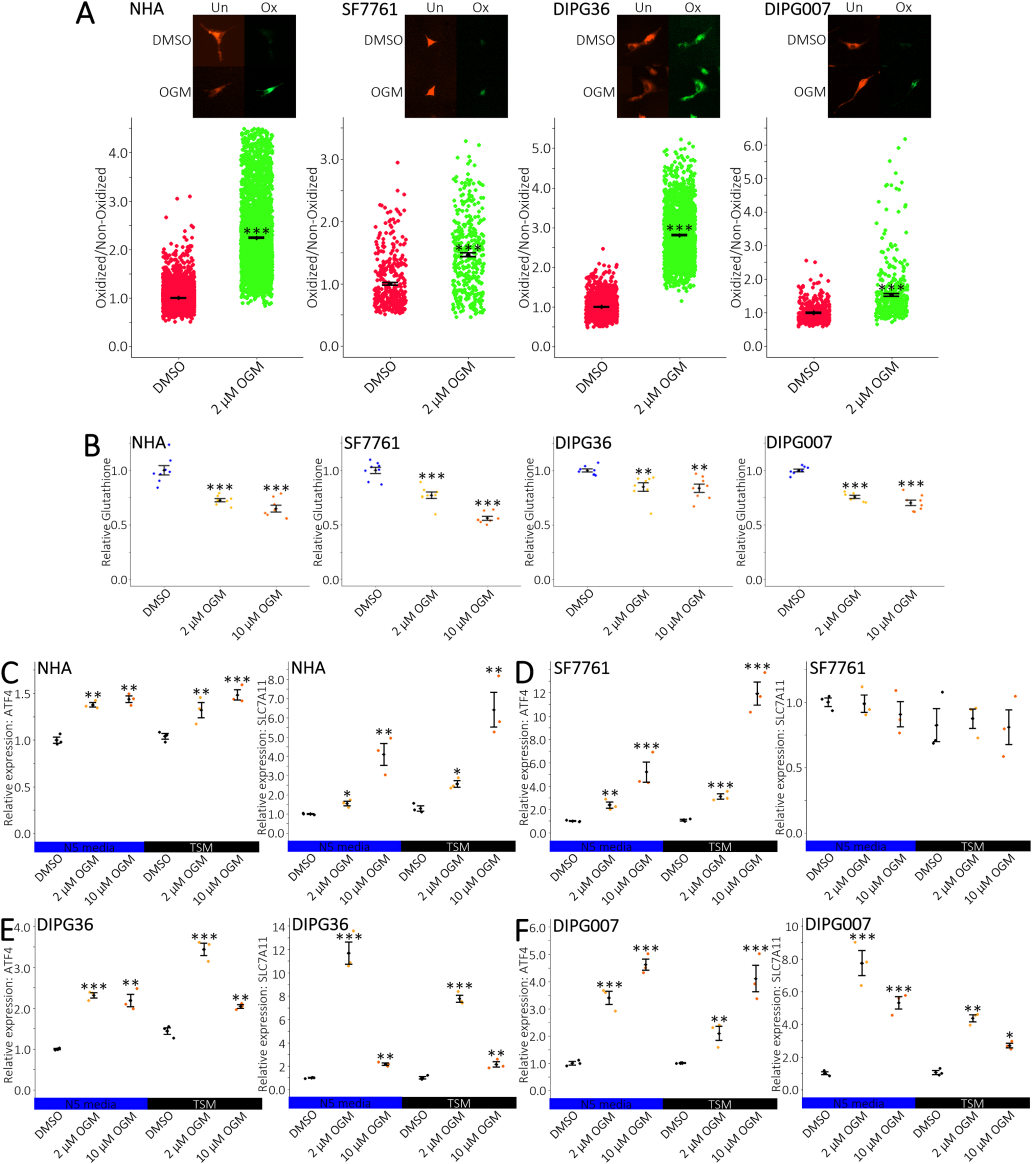
OGM induces ferroptosis in DIPGs. **(A)** BODIPY 581/591 C11 staining shows increased lipid radical formation upon treatment with OGM. Unoxidized (Un) BODIPY 581/591 C11 dye reacts with lipid radicals and becomes oxidized (Ox), shifting the signal from red to green. **(B)** Treatment with OGM significantly decreases intracellular glutathione levels. **(C)** qRT-PCR for ferroptosis reporter genes ATF4 (left) and SLC7A11 (right) in mixed and adherent cultures of NHA treated with OGM. **(D–F)** qRT-PCR for ferroptosis reporter genes ATF4 (left) and SLC7A11 (right) in mixed and adherent cultures of DIPGs treated with OGM. **(A)** *n*≥3 biological repeats with *n*≥365 aggregate cells. **(B)** *n*≥4 biological repeats with *n* = 8 technical repeats. **(C-F)** normalized to DMSO in N5 media; *n* = 3 biological repeats with *n* = 3 technical repeats. **(A)** mean +/- SEM with significance determined by two-tailed, equal variance, t-tests with **ρ<0.01, ***ρ<0.001. **(B)** mean +/- SD with significance determined by multiple two-tailed, equal variance, t-tests with Bonferroni correction. Comparisons are between control and 2 µM or 10 µM OGM with **ρ<0.0050, ***ρ<0.0005. **(C-F)** mean +/- SD with significance determined by multiple two-tailed, equal variance, t-tests with Bonferroni correction. Comparisons are between DMSO and 2 µM or 10 µM OGM for N5 media or TSM, with *ρ<0.0250, **ρ<0.0050, ***ρ<0.0005.

OGM is known to induce ferroptosis in U87MG, U138MG, A549, and PANC02 cell lines (12, 13). Therefore, we asked whether DIPGs responded similarly. Consistent with previously tested cell lines, both NHA and DIPG cells exhibited elevated levels of lipid radicals and decreased glutathione (GSH) levels in response to OGM (**Figures 3A,B****) (**12, 13). OGM promoted ferrous iron (Fe^2+^) accumulation in both NHA and DIPG cells, consistent with induction of ferroptosis (**Supplementary Figure 3****) (**12). Importantly, cell viability was rescued by the ferroptosis inhibitor liproxstatin-1 (Lip-1), but not by Z-VAD-FMK, a pan-caspase inhibitor of apoptosis (**Supplementary Figure 4 and 5**). Furthermore, treatment with OGM increased ATF4 expression, which has been reported as an essential downstream target in GPR68-mediated ferroptosis (**Figures 3C-F****) (**13). NHA, DIPG36 and DIPG007 all exhibited an increase in SLC7A11, a reporter of ferroptosis, when treated with OGM in both TSM and N5 media (**Figures 3C,E,F****) (**12, 13). However, while SF7761 cells showed an increase in ATF4, no change was observed in SLC7A11 expression regardless of media type (**Figure 3D**). Furthermore, all cell lines were sensitive to the ferroptosis inducer Erastin (ERA), suggesting a broader sensitivity of DIPGs to ferroptosis (**Supplementary Figure 6**). Taken together, these data suggest that OGM induces ferroptotic cell death in NHAs and DIPG cells.

### OGM induces ATF4 in DIPGs

**Figure 4:**
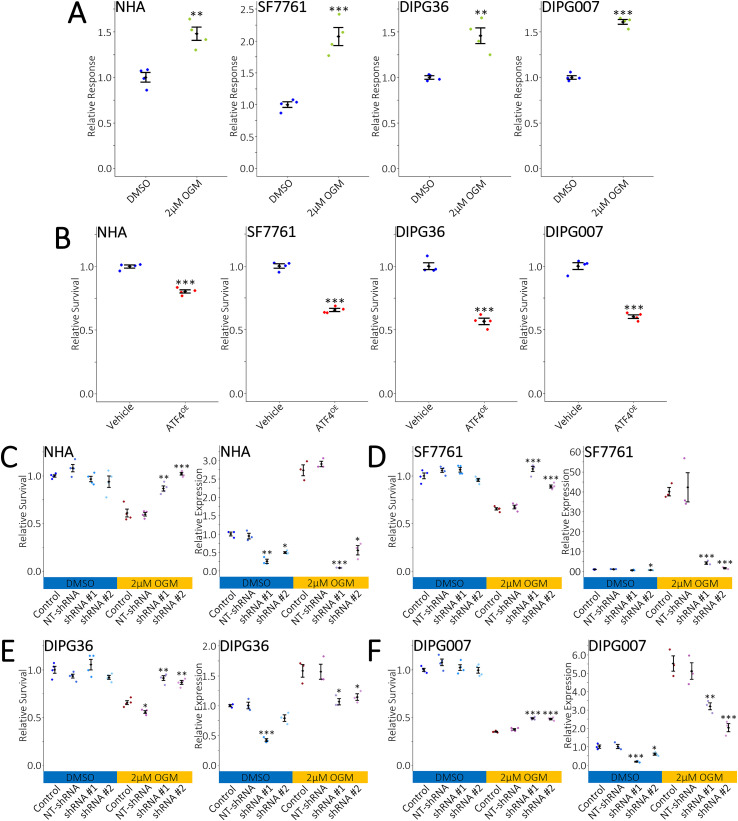
OGM induces ATF4-dependent ferroptosis in DIPGs. **(A)** OGM induces ATF4 activity in NHA and DIPG cell lines. **(B)** Overexpression of ATF4 is sufficient to induce cell death in NHA and DIPG cell lines. **(C)** shRNA-mediated ATF4 knockdown in OGM-treated NHAs rescues survival (left). Decreased ATF4 expression was verified by qRT-PCR (right). **(D-F)** shRNA-mediated ATF4 knockdown in OGM-treated DIPGs rescues survival (left). Decreased ATF4 expression was verified by qRT-PCR (right). **(A, B)** *n* = 6 biological repeats with *n* = 4 technical repeats. **(C-F)** *n* = 4 biological repeats with *n* = 4 technical repeats for survival, and *n* = 3 biological repeats with *n* = 3 technical repeats for qRT-PCR. **(A, B)** mean +/- SD with significance determined by two-tailed, equal variance, t-tests with **ρ<0.01,***ρ<0.001. **(C-F)** mean +/- SD with significance determined by multiple two-tailed, equal variance, t-tests with Bonferroni correction. Comparisons are between control and NT-shRNA, shRNA #1, or shRNA #2, with *ρ<0.01250, **ρ<0.00333, ***ρ<0.000333.

To verify the importance of ATF4 in OGM-mediated ferroptotic cell death in DIPGs we first tested an ATF4 luciferase reporter plasmid. Treatment of U87MG and U138MG GBM cell lines with OGM has been shown to increase ATF4 mRNA expression, but not activity. Here, we show an increase in luciferase expression in OGM-treated U87MG and U138MG cells, suggesting ATF4 transcriptional activity is increased (**Supplementary Figure 7**). Likewise, NHAs and all DIPG cell lines exhibited an increase in luciferase expression when treated with OGM (**Figure 4A**). Next, we tested whether ATF4 was sufficient to induce cell death in NHAs and DIPGs. Overexpression of ATF4 resulted in significant cell death in NHAs and DIPGs (**Figure 4B**). ATF4 overexpression did not promote the conversion of adherent cells into suspension cells (**Supplementary Figure 1C**). Lastly, shRNA-mediated ATF4 knockdown rescued OGM-induced cell death in NHAs and DIPG cell lines (**Figures 4C-F**). ATF4 knockdown did not alter or decrease the number of live cells in suspension for all DIPG cell lines (**Supplementary Figure 2B**). However, ATF4 knockdown induced a small increase in the number of NHA cells in suspension (**Supplementary Figure 2B**). These data suggest that OGM induces ferroptosis in NHAs and DIPGs in an ATF4-dependent manner.

## Discussion

The specific GPR68 inhibitor OGM induced ferroptosis in DIPG cells (SF7761, DIPG36, and DIPG007) comparable to GBMs (LD_50_ ≤2 µM), while NHAs were less sensitive (LD_50_ = 3.84 µM). However, GPR68 expression and sensitivity varied in different cell culture media. Knockdown of GPR68 with shRNAs validated the findings with OGM demonstrating specificity. Furthermore, OGM elevated lipid radical formation, ferrous iron accumulation, and expression of ferroptosis markers ATF4 and SLC7A11. Cell viability was rescued by the ferroptosis inhibitor Lip-1, but not by the apoptosis inhibitor Z-VAD-FMK. Similarly, treatment with the ferroptosis inducer Erastin also resulted in cell death. These data suggest GPR68 inhibition in DIPG cell linesinduces ferroptosis as it does in GBMs, A549, and PANC02 cancer cell lines (12, 13). Lastly, ATF4 overexpression drives cell death, and its knockdown rescues OGM’s effects, suggesting OGM triggers ATF4-dependent ferroptosis in DIPGs, mirroring GBM, lung, and pancreatic cancer (12, 13).

Targeting the acidified tumor microenvironment offers a promising approach for pharmacological cancer treatments as the extracellular pH sensing receptor GPR68 is salubrious to the survival of glioblastomas (13). Similar to glioblastomas, we found GPR68 also plays an essential role in the survival of DIPG cells. Herein, we demonstrated that GPR68 inhibition in DIPG cell lines induce ferroptosis, mirroring the observed effects in glioblastomas (13). Importantly, our *in vitro* findings suggest that GPR68 inhibition may be an attractive therapeutic approach for diffuse intrinsic pontine gliomas.

## Limitations

We demonstrate the effectiveness of OGM and ERA in both N5 media and TSM. However, the cell culture media does not perfectly reflect *in vivo* environments. Furthermore, we did not assess other known ferroptosis components, such as GPX4 expression and activity. While OGM has been demonstrated to be effective in A549 spheroids, PANC02 spheroids, and zebrafish xenograft models of GBM, this does not preclude the ineffectiveness of OGM on DIPGs *in vivo*.

## Methods

### Cell culture

All DIPG cells were maintained using standard protocols (see **supplemental methods**). **Figures 1**, **3C, D** were plated in working TSM or N5 media. **Figures 2**, **3A, B**, **4**, **S1, and S2** were plated in N5 media. U87MG and U138MG cells were grown in DMEM with high glucose, HEPES, and GlutaMAX (Gibco, 10564011) supplemented with 10% FBS (GeminiBio), 100 U/mL penicillin, and 100 µg/mL streptomycin (Gibco). Cells were cultured in a humidified atmosphere in an incubator maintained at 37°C and 5% CO_2_.

### LD_50_ determination

Cells were plated on two 24-well plates with working TSM and two 24-well plates with N5 media in a volume of 1 mL per well. Cells were incubated overnight at 37°C and 5% CO_2_. 1 mL of media containing 2X OGM (0.0001, 0.05, 0.1, 0.25, 0.5, 1, 2, 3, 5, 10, 20 or 40 µM) were added to wells and the cells incubated for 72 hours at 37°C and 5% CO_2_. Media was then removed from the wells and 1 mL of TrypLE express was added for 5 mins (Invitrogen, 12604-039). Cells grown in N5 were centrifuged at ≥300 g for 5 min, the supernatant removed, and the cell pellet lysed with 200 µL of 1x Passive lysis buffer (Promega, PAE1941). Cells grown in working TSM were combined with the removed working TSM media and centrifuged at ≥300 g for 5 min, the supernatant removed, and the cell pellet lysed with 200 µL of 1x Passive lysis buffer. Cells were then assessed with CellTiter-Glo (Promega, PAG7571) (20 µL lysate: 100 µL CellTiter-Glo) using LUMITRAC white-walled 96-well microplates (USA Scientific, 5665-5075). Plates were read on a GloMax-Multi+ (VWR). R-studio was used to generate a four-parameter logistic regression curve and calculate the LD_50_.

### EGFP transfections

Cells were reverse transfected in N5 media with 5 µg EGFP+ plasmid (VB900139-8384agr) per well in a 6-well plate with Lipofectamine 3000 (Invitrogen, L3000015). At 24- or 72-hours post-transfection cells were washed with DPBS (Gibco, 14190144) and the media was replaced with FluoroBrite DMEM (Gibco, A1896702). Cells were imaged and quantified on a Cytation C10 (Agilent BioTek).

### GPR68 and ATF4 overexpression assay

For GPR68 overexpression the hGPR68 (VB221110-1429uwp) and Control (VB230630-1409kyc) plasmids were obtained from VectorBuilder. For hATF4 overexpression, the hATF4 (VB230104-1203pag) and control (VB900139-8319ega) plasmids were obtained from VectorBuilder. Cells were reverse transfected in N5 media with 1.5 µg plasmid (VB900139-8384agr) per well in a 12-well plate with Lipofectamine 3000 (Invitrogen). 72 hours post-transfection cells were collected with 200 µL of 1x Passive lysis buffer. Cells were then assessed with CellTiter-Glo (20 µL lysate: 100 µL CellTiter-Glo) using LUMITRAC white-walled 96-well microplates. Plates were read on a GloMax-Multi+.

### GPR68 knockdown with shRNA

Plasmids were obtained from VectorBuilder. GPR68 shRNAs used in this paper were hGPR68-shRNA1: 5’-CCACCGTTGTCACAGACAATG-3’ (VB221221-1234czj), hGPR68-shRNA2: 5’- GAGCTGTACCATCGACCATAC-3’ (VB221221-1235jft) and non-targeting control shRNA (NT-shRNA): 5’-CCTAAGGTTAAGTCGCCCTCG-3’ (VB010000-9259tcf). ATF4 shRNAs used in this paper were hATF4-shRNA1: 5’-CATGATCCCTCAGTGCATAAA-3’ (VB230508-1473uhv), hATF4-shRNA2: 5’-CCTAGGTCTCTTAGATGATTA-3’ (VB230508-1474vhd), and non-targeting control shRNA: 5’-CCTAAGGTTAAGTCGCCCTCG-3’ (VB010000-9271yez). Cells were reverse transfected in N5 media with 1 µg plasmid per well on two 24-well plates with Lipofectamine 3000. Samples were collected with 200 µL of 1x Passive lysis buffer 72 hours post-transfection and assessed with CellTiter-Glo (20 µL lysate: 100 µL CellTiter-Glo) using LUMITRAC white-walled 96-well microplates. Plates were read on a GloMax-Multi+.

### BODIPY 581/591 C11 staining

Cells were seeded on six-well plates in N5 media and incubated overnight at 37°C and 5% CO_2_. DMSO or 2 µM OGM was then added to the cells and plates were incubated for 48 hours. Cells were then stained with BODIPY 581/591 C11 (Invitrogen, D3861) following manufacturer’s instructions for 30 min at 37°C and 5% CO_2_. Cells were washed twice with DPBS and imaged and quantified on a Cytation C10 in FluoroBrite medium.

### Glutathione assay

Cells were seeded in 75 µL N5 media per well on a black-walled 96-well tissue culture plate (VWR; 29444-008) and grown overnight at 37°C and 5% CO_2_. 75 µL of 2X DMSO or OGM was then added to each well and plates were incubated for 48 hours. Plates were then assessed using the GSH-Glo Glutathione Assay (Promega, V6911) following manufacturer’s instructions. Plates were read on a GloMax-Multi+.

### FerroOrange assay

Cells were seeded on six-well plates in N5 media and incubated overnight at 37°C and 5% CO_2_. DMSO or 2 µM OGM was then added to the cells and plates were incubated for 48 hours. Cells were then stained with FerroOrange (Dojindo Molecular Technologies Inc, F374) following manufacturer’s instructions for 30 min at 37°C and 5% CO_2_. Cells were then imaged and quantified on a Cytation C10 in FluoroBrite medium.

### Liproxstatin-1 and Z-VAD-FMK cotreatment assay

Cells were seeded on twenty four well plates in TSM media and incubated overnight at 37°C and 5% CO_2_. 2X DMSO, Lip-1, Z-VAD-FMK, and/or OGM was then added to each well and plates were incubated for 72 hours. Media was then centrifuged at ≥300 g for 5 min, the supernatant removed, and the cell pellet lysed with 200 µL of 1x Passive lysis buffer. Cells were then assessed with CellTiter-Glo (Promega, PAG7571) (20 µL lysate: 100 µL CellTiter-Glo) using LUMITRAC white-walled 96-well microplates (USA Scientific, 5665-5075). Plates were read on a GloMax-Multi+ (VWR).

### ATF4 reporter assay

The ATF4 luciferase reporter plasmid is from Addgene (plasmid #21850) (31). DIPG cells were reverse transfected in 1 mL N5 media with 1.5 µg plasmid per well in a 12-well plate with Lipofectamine 3000. U87MG and U138MG cells were reverse transfected with 3 µg plasmid in 1 mL media per well in a 12-well plate with Lipofectamine 3000. 24 hours post transfection 1 mL of 2X DMSO or OGM was added to each well and cells were incubated for 72 hours at 37°C and 5% CO_2_. Cells were collected with 200 µL of 1x Passive lysis buffer. Cells were then assessed with CellTiter-Glo (20 µL lysate: 100 µL CellTiter-Glo) and Luciferase Assay Reagent II (Promega, E1910; 20 µL lysate: 100 µL Luciferase Assay Reagent II) using LUMITRAC white walled 96-well microplates. Plates were read on a GloMax-Multi+.

### Quantitative reverse transcription PCR

Cells were grown or transfected as described with plasmids above. Total RNA was collected with TRIzol (Invitrogen, 15596026). cDNA was generated using a High-Capacity cDNA Reverse Transcription Kit with RNase Inhibitor (Applied Biosystems, 4374966). qRT-PCR was run using TaqMan Universal Master Mix II, with UNG (Applied Biosystems, 4440045) on a Quant Studio 7 Flex Real-Time PCR System (Applied Biosystems). TaqMan primers were ordered from ThermoFisher Scientific. The human primers used were GPR68 (Hs00268858s1), ATF4 (Hs00909569_g1), SLC7A11 (Hs00921938_m1), and GAPDH (Hs02786624g1).

### Trypan blue

Media was collected from samples treated as described above. Media was then mixed with trypan blue (1:1) and loaded on a Cell Counting Slide (Bio-Rad, 1450011). Live cells were assessed on a TC20 automated cell counter (Bio-Rad).

### Statistical analysis and graphs

All results are representative of at least biological triplicates. Results are given as mean ± standard deviation in **Figures 1**, **2**, **3B-D**, **4**, **S1, S2, and S3**. **Figure 3A** is given as mean ± standard error of the mean. All experiments were conducted in at least three independent biological replicates (*n*≥3), defined as experiments performed on separate days with different cell passages and freshly prepared reagents, to support robustness and reproducibility. Significant differences between the data of different groups were evaluated by Student’s t-test with *post hoc* Bonferroni correction analysis when appropriate. Significance was denoted by α levels of *<0.05, **<0.01, and ***<0.001. Significance was calculated with R 4.4.0 and graphs made in R-studio.

## Data Availability

The original contributions presented in the study are included in the article/**Supplementary Material**. Further inquiries can be directed to the corresponding authors.

## References

[1] Louis DN Perry A Reifenberger G von Deimling A Figarella-Branger D Cavenee WK . The 2016 World Health Organization classification of tumors of the central nervous system: a summary. Acta Neuropathol. (2016) 131:803–20. doi: 10.1007/s00401-016-1545-1. PMID: 27157931

[2] Schroeder KM Hoeman CM Becher OJ . Children are not just little adults: recent advances in understanding of diffuse intrinsic pontine glioma biology. Pediatr Res. (2014) 75:205–9. doi: 10.1038/pr.2013.194. PMID: 24192697

[3] Miklja Z Pasternak A Stallard S Nicolaides T Kline-Nunnally C Cole B . Molecular profiling and targeted therapy in pediatric gliomas: review and consensus recommendations. Neuro Oncol. (2019) 21:968–80. doi: 10.1093/neuonc/noz022. PMID: 30805642 PMC6682212

[4] Pellot JE De Jesus O . Diffuse intrinsic pontine glioma. In StatPearls. StatPearls Publishing. Available online at: https://www.ncbi.nlm.nih.gov/books/NBK560640/.

[5] Chen J Lin Z Barrett L Dai L Qin Z . Identification of new therapeutic targets and natural compounds against diffuse intrinsic pontine glioma (DIPG). Bioorg Chem. (2020) 99:103847. doi: 10.1016/j.bioorg.2020.103847. PMID: 32311581 PMC7260728

[6] Long W Yi Y Chen S Cao Q Zhao W Liu Q . Potential new therapies for pediatric diffuse intrinsic pontine glioma. Front Pharmacol. (2017) 8:495. doi: 10.3389/fphar.2017.00495. PMID: 28790919 PMC5525007

[7] Mohammad F Weissmann S Leblanc B Pandey DP Højfeldt JW Comet I . EZH2 is a potential therapeutic target for H3K27M-mutant pediatric gliomas. Nat Med. (2017) 23:483–92. doi: 10.1038/nm.4293. PMID: 28263309

[8] Lapin DH Tsoli M Ziegler DS . Genomic insights into diffuse intrinsic pontine glioma. Front Oncol. (2017) 7:57. doi: 10.3389/fonc.2017.00057. PMID: 28401062 PMC5368268

[9] Patel AP Tirosh I Trombetta JJ Shalek AK Gillespie SM Wakimoto H . Single-cell RNA-seq highlights intratumoral heterogeneity in primary glioblastoma. Sci (1979). (2014) 344:1396–401. doi: 10.1126/science.1254257. PMID: 24925914 PMC4123637

[10] Lauko A Lo A Ahluwalia MS Lathia JD . Cancer cell heterogeneity & plasticity in glioblastoma and brain tumors. Semin Cancer Biol. (2022) 82:162–75. doi: 10.1016/j.semcancer.2021.02.014. PMID: 33640445 PMC9618157

[11] Neitzel LR Fuller DT Williams CH Hong CC . Inhibition of GPR68 kills glioblastoma in zebrafish xenograft models. BMC Res Notes. (2024) 17:235. doi: 10.1186/s13104-024-06900-x. PMID: 39180089 PMC11342492

[12] Neitzel LR Fuller DT Cornell J Rea S de Aguiar Ferreira C Williams CH . Inhibition of GPR68 induces ferroptosis and radiosensitivity in diverse cancer cell types. Sci Rep. (2025) 15:4074. doi: 10.1038/s41598-025-88357-x. PMID: 39900965 PMC11791087

[13] Williams CH Neitzel LR Cornell J Rea S Mills I Silver MS . GPR68-ATF4 signaling is a novel prosurvival pathway in glioblastoma activated by acidic extracellular microenvironment. Exp Hematol Oncol. (2024) 13:13. doi: 10.1186/s40164-023-00468-1. PMID: 38291540 PMC10829393

[14] Wei J Wang L Mao Z Zhang P . Targeting GPR68 alleviates inflammation and lipid accumulation in metabolic dysfunction-associated steatohepatitis. Biol (Basel). (2026) 15:233. doi: 10.3390/biology15030233. PMID: 41677704 PMC12896580

[15] Ludwig M Vanek M Guerini D Gasser JA Jones CE Junker U . Proton-sensing G-protein-coupled receptors. Nature. (2003) 425:93–8. doi: 10.1038/nature01905. PMID: 12955148

[16] Kondo A Yamamoto S Nakaki R Shimamura T Hamakubo T Sakai J . Extracellular acidic pH activates the sterol regulatory element-binding protein 2 to promote tumor progression. Cell Rep. (2017) 18:2228–42. doi: 10.1016/j.celrep.2017.02.006. PMID: 28249167

[17] Roma-Rodrigues C Mendes R Baptista PV Fernandes AR . Targeting tumor microenvironment for cancer therapy. Int J Mol Sci. (2019) 20:840. doi: 10.3390/ijms20040840. PMID: 30781344 PMC6413095

[18] Hunter A Hendrikse A Renan M Abratt R . Does the tumor microenvironment influence radiation-induced apoptosis? Apoptosis. (2006) 11:1727–35. doi: 10.1007/s10495-006-9789-1. PMID: 16927015

[19] Bailey KM Wojtkowiak JW Hashim AI Gillies RJ . Targeting the metabolic microenvironment of tumors. (2012) 65:63–107. doi: 10.1016/B978-0-12-397927-8.00004-X PMC3796340 22959024

[20] Worsley CM Veale RB Mayne ES . The acidic tumour microenvironment: manipulating the immune response to elicit escape. Hum Immunol. (2022) 83:399–408. doi: 10.1016/j.humimm.2022.01.014. PMID: 35216847

[21] Kato Y Ozawa S Miyamoto C Maehata Y Suzuki A Maeda T . Acidic extracellular microenvironment and cancer. Cancer Cell Int. (2013) 13:89. doi: 10.1186/1475-2867-13-89. PMID: 24004445 PMC3849184

[22] Sutoo S Maeda T Suzuki A Kato Y . Adaptation to chronic acidic extracellular pH elicits a sustained increase in lung cancer cell invasion and metastasis. Clin Exp Metastasis. (2020) 37:133–44. doi: 10.1007/s10585-019-09990-1. PMID: 31489536 PMC7007909

[23] Justus CR Dong L Yang LV . Acidic tumor microenvironment and pH-sensing G protein-coupled receptors. Front Physiol. (2013) 4:354. doi: 10.3389/fphys.2013.00354. PMID: 24367336 PMC3851830

[24] Boedtkjer E Pedersen SF . The acidic tumor microenvironment as a driver of cancer. Annu Rev Physiol. (2020) 82:103–26. doi: 10.1146/annurev-physiol-021119-034627. PMID: 31730395

[25] Cornell J Rea S Neitzel LR Williams CH Hong CC . Proton sensing GPCR’s: The missing link to Warburg’s oncogenic legacy? J Cancer Biol. (2024) 5:65–75. doi: 10.46439/cancerbiology.5.066. PMID: 39641117 PMC11619763

[26] Chung C Sweha SR Pratt D Tamrazi B Panwalkar P Banda A . Integrated metabolic and epigenomic reprograming by H3K27M mutations in diffuse intrinsic pontine gliomas. Cancer Cell. (2020) 38:334–349.e9. doi: 10.1016/j.ccell.2020.07.008. PMID: 32795401 PMC7494613

[27] Autry AW Park I Kline C Chen HY Gordon JW Raber S . Pilot study of hyperpolarized ^13^ C metabolic imaging in pediatric patients with diffuse intrinsic pontine glioma and other CNS cancers. Am J Neuroradiol. (2021) 42:178–84. doi: 10.3174/ajnr.a6937. PMID: 33272950 PMC7814790

[28] Doan NB Nguyen HS Alhajala HS Jaber B Al-Gizawiy MM Ahn EE . Identification of radiation responsive genes and transcriptome profiling via complete RNA sequencing in a stable radioresistant U87 glioblastoma model. Oncotarget. (2018) 9:23532–42. doi: 10.18632/oncotarget.25247. PMID: 29805753 PMC5955095

[29] Röttinger EM Mendonca M . Radioresistance secondary to low ph in human glial cells and Chinese hamster ovary cells. Int J Radiat Oncol Biol Phys. (1982) 8:1309–14. doi: 10.1016/0360-3016(82)90580-6 7141909

[30] Wang T Zhou G He M Xu Y Rusyniak WG Xu Y . GPR68 is a neuroprotective proton receptor in brain ischemia. Stroke. (2020) 51:3690–700. doi: 10.1161/strokeaha.120.031479. PMID: 33059544 PMC7678672

[31] Harding HP Novoa I Zhang Y Zeng H Wek R Schapira M . Regulated translation initiation controls stress-induced gene expression in mammalian cells. Mol Cell. (2000) 6:1099–108. doi: 10.1016/s1097-2765(00)00108-8. PMID: 11106749

