# Supplementary material for: Inhibition of GPR68 induces ferroptosis in diffuse intrinsic pontine gliomas
Source: Front Oncol. 2026 May 13;16:1808752. doi: 10.3389/fonc.2026.1808752 (PMC13212223; doi:10.3389/fonc.2026.1808752)
Supplement: Supplementary file 1 [file DataSheet1.pdf]

A

	DMSO			OGM			T-test
NHA	1	+/-	0.045	0.723	+/-	0.040	2.87E-03
SF7761	1	+/-	0.024	1.053	+/-	0.014	5.34E-02
DIPG36	1	+/-	0.027	0.235	+/-	0.003	2.32E-06
DIPG007	1	+/-	0.147	1.259	+/-	0.192	2.05E-01

B

	ORF stuffer			hGPR68			T-test
NHA	1	+/-	0.044	1.007	+/-	0.244	9.68E-01
SF7761	1	+/-	0.094	1.126	+/-	0.160	3.90E-01
DIPG36	1	+/-	0.090	0.945	+/-	0.023	4.54E-01
DIPG007	1	+/-	0.077	1.094	+/-	0.067	2.61E-01

C

	ORF stuffer			hATF4			T-test
NHA	1	+/-	0.010	0.736	+/-	0.014	2.47E-05
SF7761	1	+/-	0.040	0.704	+/-	0.171	7.54E-02
DIPG36	1	+/-	0.073	0.726	+/-	0.180	1.18E-01
DIPG007	1	+/-	0.048	0.365	+/-	0.017	5.86E-05

Supplemental Figure 1: Treatments did not promote conversion from adherent to suspension cells. (A) Treatment of cells with DMSO or OGM in N5 media. The number of suspension cells did not change significantly in the SF7761 or DIPG007 cell lines. The number of NHA and DIPG36 suspension cells decreased significantly. **(B)** Transfection of cells in N5 media with control or hGPR68 overexpression plasmid did not change the number of suspension cells in any line. **(C)** Transfection of cells in N5 media with control or hATF4 overexpression plasmid did not change the number of suspension cells in SF7761 or DIPG36. The number of NHA and DIPG007 suspension cells decreased significantly. **(A)**, **(B)**, and **(C)** *n*=3 biological repeats. Mean +/- SD with significance determined by two-tailed, equal variance, t-tests.

A

	Control	NT-shRNA	T-test	shRNA #1	T-test	shRNA #2	T-test
NHA	1 +/- 0.045	0.792 +/- 0.039	7.87E-03	0.741 +/- 0.014	1.47E-03	0.867 +/- 0.011	1.53E-02
SF7761	1 +/- 0.068	0.844 +/- 0.023	3.71E-02	0.532 +/- 0.073	2.70E-03	0.463 +/- 0.047	7.84E-04
DIPG36	1 +/- 0.023	0.877 +/- 0.047	2.96E-02	0.826 +/- 0.135	1.46E-01	0.688 +/- 0.076	5.26E-03
DIPG007	1 +/- 0.013	0.973 +/- 0.036	3.75E-01	0.572 +/- 0.028	4.09E-05	0.604 +/- 0.048	3.71E-04

B

	Control	NT-shRNA	T-test	shRNA #1	T-test	shRNA #2	T-test
NHA	1 +/- 0.034	0.916 +/- 0.025	4.85E-02	1.142 +/- 0.016	5.91E-03	1.136 +/- 0.016	6.77E-03
SF7761	1 +/- 0.034	0.987 +/- 0.015	6.51E-01	1.101 +/- 0.042	5.70E-02	0.980 +/- 0.016	4.99E-01
DIPG36	1 +/- 0.010	1.032 +/- 0.076	5.82E-01	0.843 +/- 0.059	2.11E-02	0.906 +/- 0.036	2.39E-02
DIPG007	1 +/- 0.074	1.157 +/- 0.097	1.42E-01	1.063 +/- 0.016	3.03E-01	0.873 +/- 0.049	1.14E-01

Supplemental Figure 2: shRNA did not promote conversion from adherent to suspension cells. (A) Cells transfected with shRNA against hGPR68 in N5 media. The number of suspension cells was unchanged or decreased significantly in all cell lines. **(B)** Cells transfected with shRNA against hATF4 in N5 media. The number of suspension cells was unchanged or decreased significantly in SF7761, DIPG36, and DIPG007 cell lines. NHA suspension cells increased slightly when transfected with shRNA #1 or shRNA #2 but did not with control shRNA. **(A)** and **(B)** *n*=3 biological repeats. Mean +/- SD with significance determined by multiple two-tailed, equal variance, t-tests. Control shRNA, shRNA #1, and shRNA #2 were compared to control cells. Adjusted α-levels ($\rho<0.05$, 0.01, 0.001) are 0.01250, 0.00333, and 0.000333 respectively.

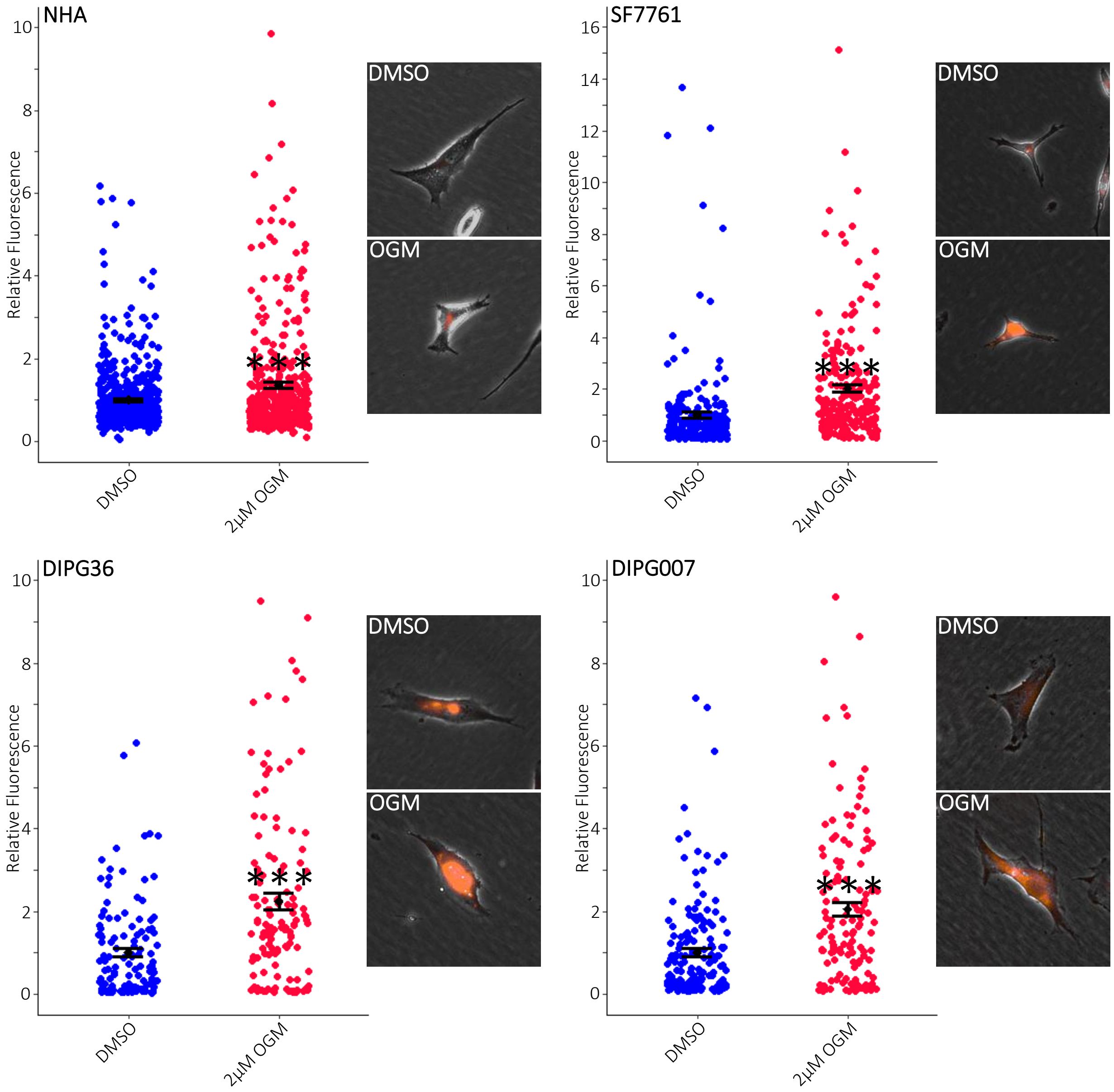

Supplemental Figure 3: OGM upregulates ferrous iron in NHA and DIPG cell lines. FerroOrange staining of cells treated with DMSO or OGM in N5 media. (Left) Quantification of ferrous (Fe^{2+}) iron. (Right) Example images of stained cells. Data aggregated from *n*=4 biological repeats. Mean +/- SEM, normalized to DMSO, with significance determined by two-tailed, equal variance, t-tests. ***ρ<0.001.

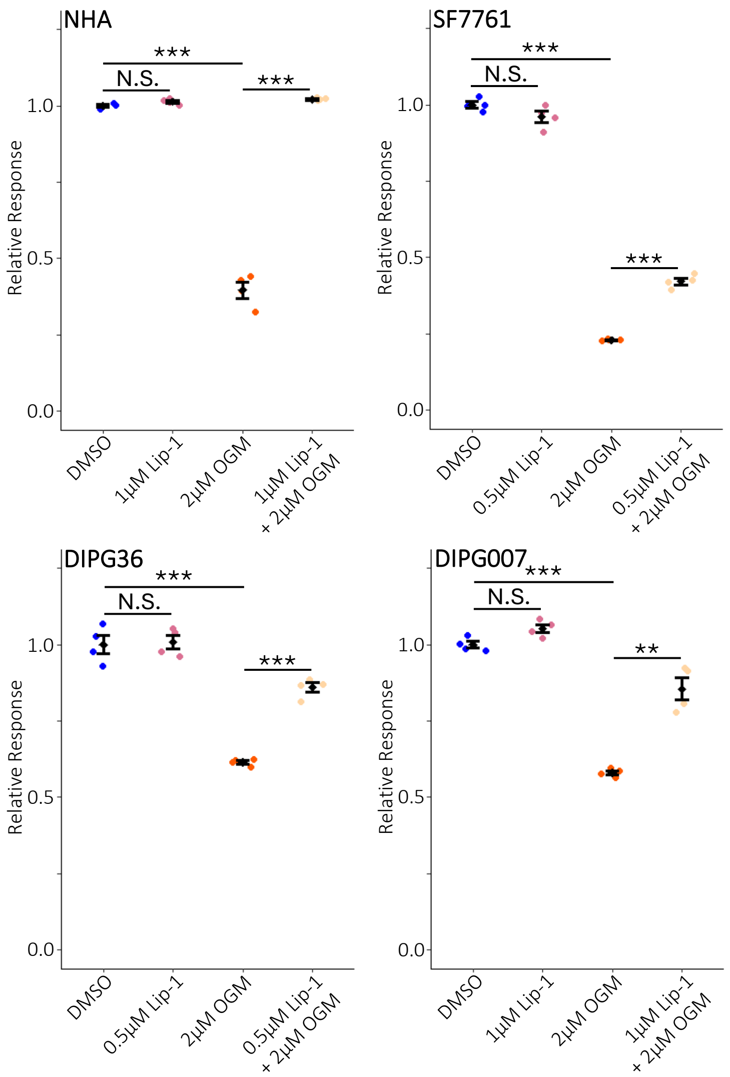

Supplemental Figure 4: Liproxstatin-1 rescues OGM mediated ferroptosis. Treatment of cells with DMSO, Lip-1, OGM, or cotreatment with Lip-1 and OGM in TSM. *n*≥4 biological repeats with *n*=4 technical repeats. Mean +/- SD, normalized to DMSO, with significance determined by two-tailed, equal variance, t-tests with Bonferroni correction. Comparisons are between DMSO and OGM or Lip-1, and OGM and co-treatment, with **p<0.0033 and ***ρ<0.00033.

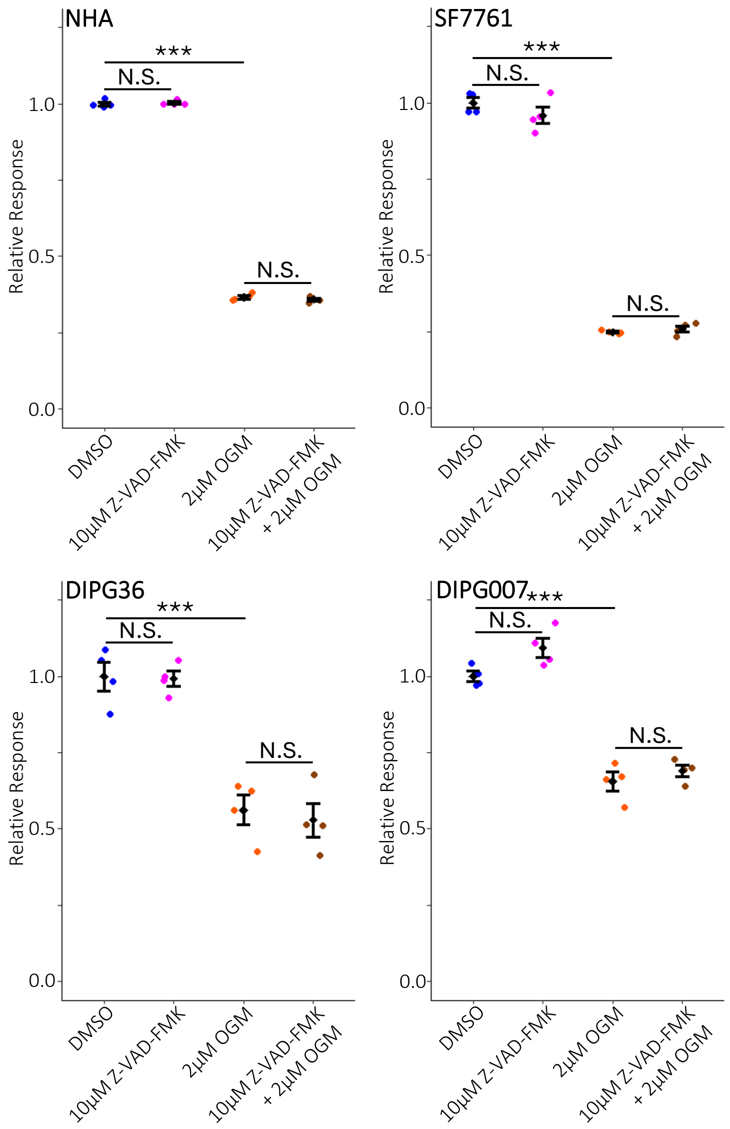

Supplemental Figure 5: Z-VAD-FMK does not rescue OGM mediated ferroptosis. Treatment of cells with DMSO, Z-VAD-FMK, OGM, or cotreatment with Z-VAD-FMK and OGM in TSM. *n*≥4 biological repeats with *n*=4 technical repeats. Mean +/- SD, normalized to DMSO, with significance determined by two-tailed, equal variance, t-tests with Bonferroni correction. Comparisons are between DMSO and OGM or Z-VAD-FMK, and OGM and co-treatment, with ***ρ<0.00033.

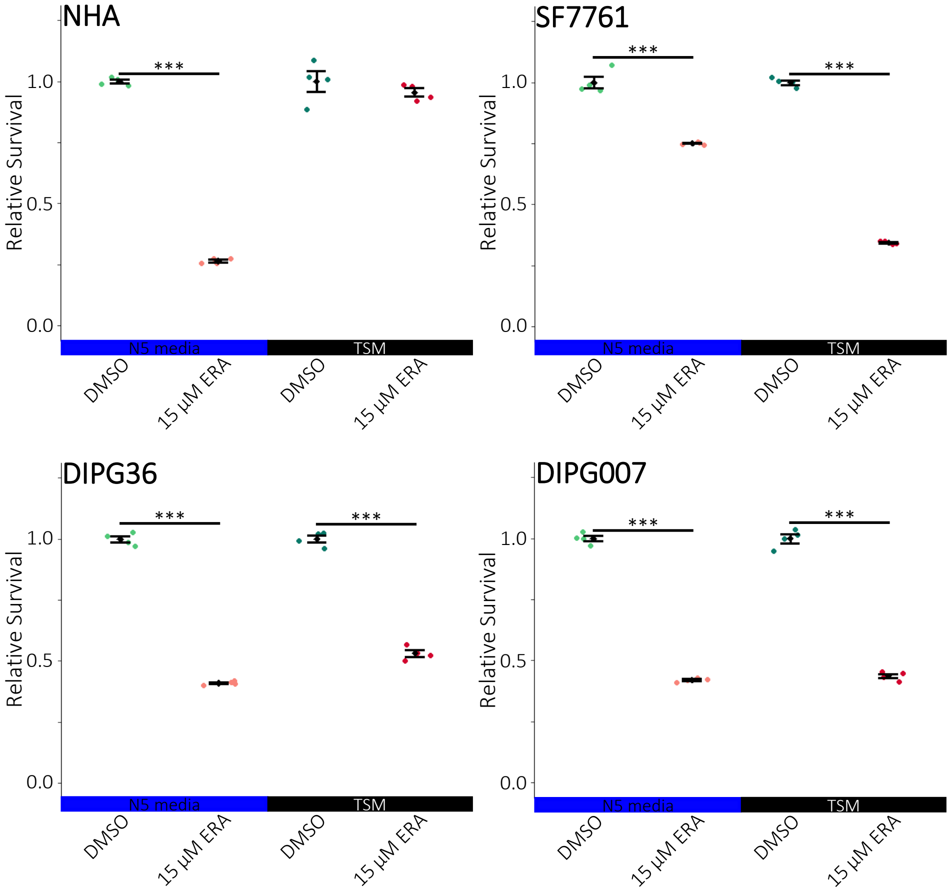

Supplemental Figure 6: Erastin induces ferroptosis in NHA and DIPG cell lines. Treatment of cells with DMSO or ERA in N5 media or TSM. *n*=3 biological repeats with *n*=4 technical repeats. Mean +/- SD, normalized to DMSO, with significance determined by two-tailed, equal variance, t-tests. ***$\rho<0.001$.

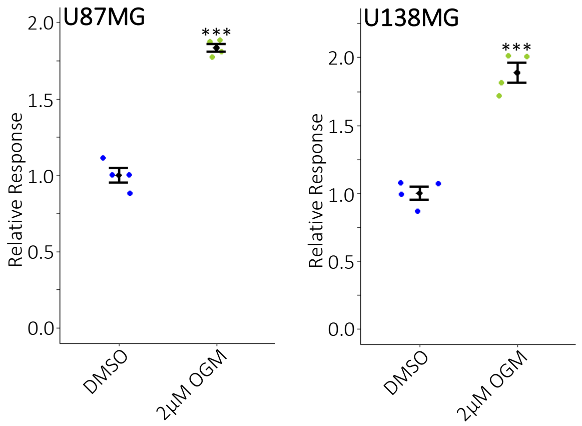

Supplemental Figure 7: OGM induces ATF4 in glioblastoma cell lines.

OGM induces ATF4 activity in U87MG (left) and U138MG (right) cell lines. *n*=3 biological repeats with *n*=4 technical repeats. Mean +/- SD with significance determined by two-tailed, equal variance, t-tests. ***ρ<0.001.
