# Supplementary material for: Inhibition of GPR68 induces ferroptosis in diffuse intrinsic pontine gliomas
Source: Front Oncol. 2026 May 13;16:1808752. doi: 10.3389/fonc.2026.1808752 (PMC13212223; doi:10.3389/fonc.2026.1808752)
Supplement: Supplementary file 2 [file DataSheet2.pdf]

Cell culture

All medias were prepared in a biological safety cabinet using sterile technique. Cells were incubated at 37°C and 5% CO_2 in a filter cap T75 tissue culture flask. Media is replaced every two days or sooner if the media becomes yellow.

Tumor Stem Medium (TSM) Base

Tumor Stem Medium (TSM) Base			
Reagent	Volume (mL)	Catalog #	Vendor
Neurobasal-A Medium (1X)	250	10888-022	Invitrogen
D-MEM/F-12 (1X; 1:1 ratio)	250	11330-032	Invitrogen
HEPES Buffer Solution (1M)	5	15630-080	Invitrogen
MEM Sodium Pyruvate Solution 100mM (100X)	5	11360-070	Invitrogen
MEM Non-Essential Amino Acids Solution 10mM (100X)	5	11140-050	Invitrogen
GlutaMAX-I Supplement	5	35050-061	Invitrogen
Antibiotic-Antimycotic (100X)	5	15240-096	Invitrogen

Note: All reagents are solutions

Note: TSM base was passed through a 0.22 µm filter (Corning; 431205) and stored at 4°C.

Working TSM

Working TSM is made fresh on the day of use. The EGF, FGF, PDGF-AA, PDGF-BB and heparin were added to the TSM Base and passed through a 0.22 µm filter (Millipore; SCGP00525).

Working TSM (1x)				
Reagent	Volume	Stock Concentration (1000x)	Catalog #	Vendor
TSM Base	50 mL	-	-	-
B-27 Supplement Minus Vitamin A (50X)	1 mL	-	12587-010	Invitrogen
H-EGF	50 µL	20 µg/mL	100-26	Shenandoah Biotech
H-FGF-basic-154	50 µL	20 µg/mL	100-146	Shenandoah Biotech
H-PDGF-AA	25 µL	20 µg/mL	100-16	Shenandoah Biotech
H-PDGF-BB	25 µL	20 µg/mL	100-18	Shenandoah Biotech
Heparin Solution, 0.2%	50 µL	2 mg/mL	07980	StemCell Technologies, Inc.

Note: EGF, FGF, PDGF-AA, PDGF-BB and heparin stocks are frozen with liquid nitrogen and stored at-80°C

N5 Media

To induce adherent cultures DIPGs are cultured in N5 media.

N5 Media				
Reagent	Volume	Stock Concentration (1000x)	Catalog #	Vendor
Working TSM (1x)	45 mL	-	-	-
Fetal Bovine Serum (FBS)	5 mL	-	A5670701	Gibco

Feeding cells

1) Prepare fresh Working TSM in a 50mL conical tube
2) Warm media in a 37°C water bath for 10 min or until warm
3) Transfer half of the media from the T75 to a 15 mL conical
4) Centrifuge the 15 mL conical at ≥300 g for 5 min
5) Remove the medium (supernatant)
6) Resuspend the cells pellet in fresh pre-warmed media
7) Transfer the resuspended cells back into the original tissue culture flask
8) Return cells to the incubator

Passaging and plating cells

1) Pre-warm HBSS (Fisher, MT21022CV) and Working TSM media
    a. (Optional) Pre-warm N5 media
2) Transfer all media from the T75 to an appropriate conical
3) Quickly add 7 mL of TrypLE express (Invitrogen, 12604-039) to the empty T75
4) Add 70 µL of 10 mg/mL DNase (Worthington, LS002007) solution
5) Incubate the flask for 5 min at 37ºC
6) Concurrently with step 5, centrifuge the tube at ≥300 g for 5 min
7) Discard supernatant from the conical tube
8) Transfer the 7 mL TrypLE + cells mixture to the conical tube with the cell pellet
9) Triturate the pellet in the 7 mL TrypLE + cells mixture
10) Add an additional fresh 5 mL of TrypLE express to the T75
11) Add 50 uL of 10 mg/mL DNase to the T75
12) Pipette up and down the TrypLE in the T75 to release the remaining adherent cells
13) Transfer the 5 mL TrypLE to the conical tube (total volume of 12 mL)
14) Gently rotate the conical tube in a nutator for 5-10 min at 37ºC
15) Triturate the cells again with a 10 mL pipette to dissociate the cells
16) Add 22 mL of pre-warmed to 37ºC HBSS to the cell mixture
17) Mix the solution
18) Filter the mixture through a 40 µm membrane cell strainer (Fisher, 08771)
19) Centrifuge at ≥300 g for 7 min
20) Aspirate the supernatant

For passaging

21) Resuspend cells in fresh working TSM
22) Plate in new filter cap T75 tissue culture flask and store at 37ºC and 5% CO_2

For plating

21) Resuspend cells in fresh pre-warmed to 37ºC HBSS
22) Count cells
23) Centrifuge the desired volume/cell number
24) Resuspend in fresh working TSM or N5 media
25) Distribute to plate
